# Structural Basis of the Intracellular Sorting of the SNARE VAMP7 by the AP3 Adaptor Complex

**DOI:** 10.1016/j.devcel.2012.01.018

**Published:** 2012-05-15

**Authors:** Helen M. Kent, Philip R. Evans, Ingmar B. Schäfer, Sally R. Gray, Christopher M. Sanderson, J. Paul Luzio, Andrew A. Peden, David J. Owen

**Affiliations:** 1 Medical Research Council Laboratory of Molecular Biology, Hills Road, Cambridge CB2 2QH, UK; 2 Cambridge Institute for Medical Research and Department of Clinical Biochemistry, University of Cambridge, Addenbrooke's Hospital, Hills Road, Cambridge CB2 0XY, UK; 3 Medical Research Council Rosalind Franklin Centre for Genomics Research, Hinxton, Cambridge CB10 1SB, UK

## Abstract

VAMP7 is involved in the fusion of late endocytic compartments with other membranes. One possible mechanism of VAMP7 delivery to these late compartments is via the AP3 trafficking adaptor. We show that the linker of the δ-adaptin subunit of AP3 binds the VAMP7 longin domain and determines the structure of their complex. Mutation of residues on both partners abolishes the interaction in vitro and in vivo. The binding of VAMP7 to δ-adaptin requires the VAMP7 SNARE motif to be engaged in SNARE complex formation and hence AP3 must transport VAMP7 when VAMP7 is part of a *cis*-SNARE complex. The absence of δ-adaptin causes destabilization of the AP3 complex in mouse *mocha* fibroblasts and mislocalization of VAMP7. The mislocalization can be rescued by transfection with wild-type δ-adaptin but not by δ-adaptin containing mutations that abolish VAMP7 binding, despite in all cases intact AP3 being present and LAMP1 trafficking being rescued.

## Graphical abstract

**Graphical Abstract:**

**Figure:**
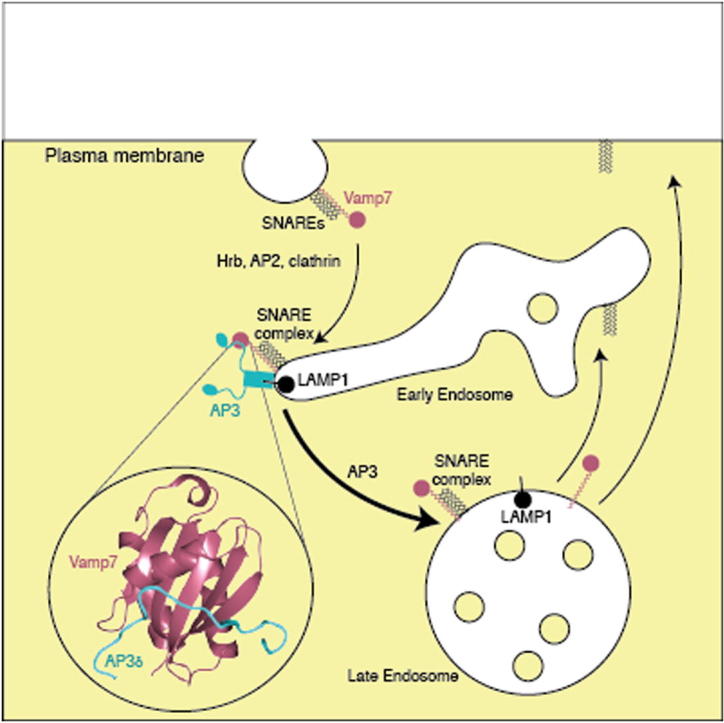

**Highlights:**

► AP3-containing vesicles transport VAMP7 and LAMP1 from early to late endosomes ► The ear/trunk linker of the AP3 δ-subunit binds in a groove on a VAMP7 longin domain ► Binding of VAMP by AP3 can only occur when VAMP is part of a *cis*-SNARE complex ► VAMP7 is not required for the fusion of AP3-containing vesicles with late endosomes

## Teaser

VAMP7 SNARE complexes on late endosomes determine the specificity of membrane fusion there. But how is VAMP7 localization maintained? Kent et al. define the structure of and requirement for VAMP7 interactions with the cargo adaptor AP3. AP3 uses distinct surfaces for VAMP7 versus other cargoes to recycle postfusion *cis*-SNARE complexes.

## Introduction

SNAREs (soluble NSF [N-ethylmaleimide-sensitive factor] attachment receptors) are mainly small (100–300 residue), C-terminally membrane anchored proteins (Hong, 2005; Jahn and Scheller, 2006). There are at least 38 SNAREs encoded in the human genome and all contain at least one characteristic SNARE motif. This is an evolutionarily conserved stretch of 60–70 residues in which hydrophobic side chain amino acids are arranged in heptad repeats. SNAREs are classified as R (also termed v-) or Q (also termed t-) depending on the identity of the residue at the central position (position 0), of the SNARE motif. Four of these SNARE motifs from SNAREs in two different membranes form very tight complexes in a limited number of combinations, bringing the two membranes into close apposition so allowing the two membranes to fuse. Thus the SNAREs provide much of the specificity as well as energy to the fusion of membranes in vivo. In addition to their SNARE motifs, most SNAREs (with the exception of SNAP23, SNAP25, and SNAP29) possess an N-terminal region that can vary from a 10–30 short stretch of unstructured residues to an approximately 100 residue folded domain (for review, see Hong, 2005; Jahn and Scheller, 2006).

The central role of SNAREs in defining the specificity of membrane fusion requires them to be placed correctly in both the vesicle membrane and in the target membrane, and hence it is logical to suppose that their membrane localization, which must be defined by their trafficking itinerary, is actively controlled. The selective sorting of transmembrane proteins into transport vesicles or tubules is effected by the direct interaction between the desired cargo and a component of the coat that surrounds the vesicle/tubule. The first characterized examples of such interactions for SNAREs were those of the SNAREs involved in ER to Golgi transport by the COPII coat (Mancias and Goldberg, 2007; Mossessova et al., 2003). In post-Golgi trafficking, conventional transmembrane protein cargo must be actively and specifically sorted into the various vesicular/tubular carriers used for the various different trafficking routes. These carriers must also contain sufficient amounts of the correct SNAREs to allow them to fuse with their desired target membrane and may also be needed to carry other SNAREs back to their required membrane localization. In addition to the interaction of VAMP4 through its standard ExxxLL motif with the clathrin adaptor AP1 (Peden et al., 2001), three examples of SNARE selection in these post-Golgi steps have been described in detail (Miller et al., 2007, 2011; Pryor et al., 2008). Both interactions are with specialized clathrin adaptors that do not bind standard cargo (containing YxxΦ, [ED]xxxL[LI], or FxNPxY motifs), thus allowing the interactions to occur in parallel with standard cargo selection, without competing with it.

VAMP7 (also termed Ti-VAMP) (Advani et al., 1998; Galli et al., 1998) is one of the largest R-SNAREs, having a 120 residue N-terminal longin domain (Pryor et al., 2008; Rossi et al., 2004). It is involved in the fusion of lysosomes, late endosomes, and compartments derived from them with a number of target membranes including the plasma membrane and endosomes (for a comprehensive review of VAMP7 function see (Chaineau et al., 2009)). Yeast two-hybrid (Y2H) screening performed by the Galli laboratory (Martinez-Arca et al., 2003) and by us (Pryor et al., 2008) indicated the delta subunit of AP3 as a major binding partner of the VAMP7 longin domain. AP3 (subunits δ, β3, μ3, σ3) is one of the family of heterotetrameric clathrin adaptors that in mammalian cells has been localized mainly to tubulovesicular, early-sorting/recycling endosomes with a minor location at the *trans*-Golgi network (TGN) (Peden et al., 2004; Simpson et al., 1996; Theos et al., 2005). Tubular recycling endosomes are often located close to the TGN and there are traffic pathways between them (Chapuy et al., 2008; Maxfield and McGraw, 2004; Nokes et al., 2008). AP3 is thought to be involved in the transport of proteins to late endosomes/lysosomes (Dell'Angelica et al., 1999; Le Borgne et al., 1998), in the biogenesis of specialized lysosome-related compartments including melanosomes and platelet granules and also in the biogenesis of synaptic vesicles (for review, see Dell'Angelica, 2009). Its two large subunits (β3 and δ) are both comprised of a 70 kDa helical solenoid trunk joined by a natively unstructured linker to a 30 kDa appendage. In at least some of its trafficking roles it must use clathrin since it contains a highly conserved clathrin box in its β3 linker (Dell'Angelica et al., 1998; Peden et al., 2002).

Here, we use a combination of X-ray crystallography, protein interaction assays and in vivo studies making use of cell lines generated from δ-adaptin-deleted *mocha* mouse fibroblasts to investigate the molecular nature and cellular role of the VAMP7/δ-adaptin interaction.

## Results

### VAMP7 Longin Domain Binds Directly to the δ-Adaptin Linker Region

**Figure 1:**
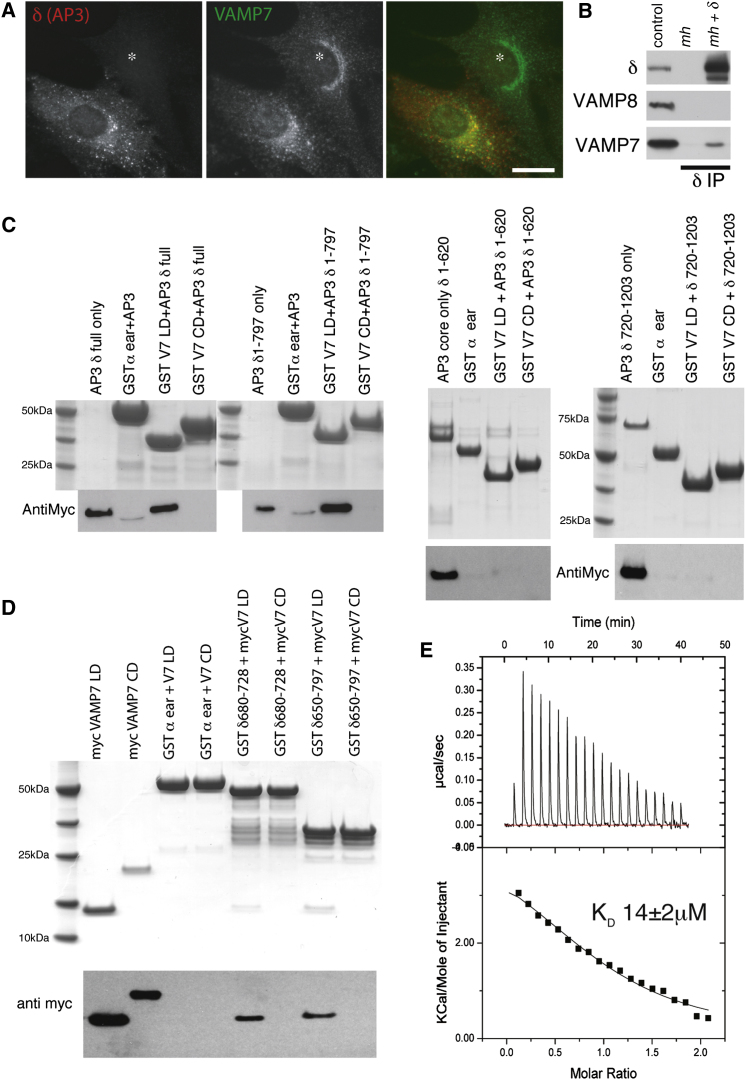
The δ-Adaptin Linker Binds to the Longin Domain Only of VAMP7 (A) *Mocha* fibroblasts were mixed with cells expressing δ^wt^ adaptin and then fixed and stained with antibodies to δ-adaptin and VAMP7. Asterisk indicates nontransduced cell. Scale bar, 20 μm. (B) *Mocha* fibroblasts were retrovirally transduced with δ^wt^ adaptin and native immunoprecipitations performed with antibodies to δ-adaptin. The immunoprecipitations were immunoblotted for the δ subunit of the AP-3 complex and the post-Golgi R-SNAREs VAMP8 (negative control) and VAMP7. As a positive control for the immunoblotting 3% of the *mocha +* δ^wt^ detergent extract was loaded (control). (C) In GST pull-down experiments, GSTVAMP7 longin domain (LD) but not cytoplasmic domain (CD) or GST α-ear bind to recombinant AP3 containing either full-length δ-adaptin (residues 1–1,203) or δ-adaptin trunk+hinge (residues 1–797) but not to AP3 containing δ-adaptin trunk (residues 1–620) or the δ-adaptin appendage (residues 720–1203). Top panels Coomassie blue staining and bottom panels western blots for the Myc tag at the C terminus of μ3 adaptin. (D) GST δ-adaptin (650–797) and GST δ-adaptin (680–728) but not GSTα-ear bind to His_6_MycVAMP7LD but not His_6_MycVAMPCD as detected by western blotting with an anti-Myc antibody. Top panels Coomassie blue staining and bottom panels western blots for the Myc tag on VAMP7 constructs. (E) VAMP7LD binds to GSTδ-adaptin (680–728) with a K_D_ of 14 ± 2 μM by isothermal titration calorimetry. See also Figure S1.

The absence of δ-adaptin, in *mocha* cells leads to the destabilization and degradation of all the other AP3 subunits and causes an increase in trafficking via the cell surface of lysosomal membrane proteins such as LAMPI and CD63 (Kantheti et al., 1998; Peden et al., 2002; Rous et al., 2002). This *mocha* cell phenotype can be rescued by re-expressing δ-adaptin, which leads to the stabilization of the other subunits (Martinez-Arca et al., 2003; Peden et al., 2004). In untransduced *mocha* cells, VAMP7 is localized to a reticular, perinuclear compartment (Figure 1A) that is positive for TGN markers (see below), whereas in cells transduced with wt δ-adaptin VAMP7 has its expected localization in late endosomes/lysosomes (Advani et al., 1998; Chaineau et al., 2009). These data suggested that AP3 is involved in the trafficking of VAMP7 to late endocytic compartments. The observation that VAMP7 but not other VAMPs can be detected in a native immunoprecipitation with an antibody against δ-adaptin from wt δ-adaptin transduced *mocha* cell cytosol indicates that AP3 interacts either directly or indirectly with VAMP7 (Figure 1B).

A direct interaction between VAMP7 and AP3 is consistent with the observation that binding between δ-adaptin and both the longin domain and full-length cytoplasmic domain of VAMP7 was observed in Y2H screening, (Martinez-Arca et al., 2003). In a Y2H screen carried out at a similar time to (Martinez-Arca et al., 2003) in our laboratory, however, an interaction with δ-adaptin was detected only with the longin domain (residues 1–120) of VAMP7 (see Figure S1 available online). In an equivalent screen using the full-length cytoplasmic domain of VAMP7 we obtained no δ-adaptin clones but, as in (Burgo et al., 2009; Martinez-Arca et al., 2003), we did identify SNAP-29 (1 out of 14 positive clones) and Varp (11 out of 14 positive clones), thus validating this screen. Glutathione S-transferase (GST) pull-down experiments using recombinant bacterially expressed VAMP7 domains and AP3 complexes (Figure 1C) confirmed that the interaction with AP3 did indeed only occur with the longin domain and not with the full cytosolic domain of VAMP7.

Thirty-seven δ−adaptin clones were isolated from our Y2H screen with the VAMP7 longin domain (Pryor et al., 2008) and the binding site for the longin domain, predicted from the overlap of these clones, was mapped to residues 651–797, which is the majority of the δ-adaptin linker domain that separates its appendage domain from its trunk domain and includes the region proposed in (Martinez-Arca et al., 2003). In agreement with the Y2H mapping, both recombinant AP3 containing full-length δ-adaptin (residues 1–1,203) and that containing δ-adaptin trunk+hinge (residues 1–797) but not that containing only the δ-adaptin trunk (residues 1–620) nor the δ-adaptin appendage domain alone (residues 720–1,203) bound to the GST-VAMP7 longin domain (Figure 1C). Further, GST appended with residues 650–797 of δ-adaptin bound only to the VAMP7 longin domain and not to the full-length cytosolic domain (Figure 1D). Inspection of the sequence of the δ-adaptin linker suggested that the region between residues 680 and 730 was the most likely candidate for VAMP7 binding (Pryor et al., 2008; Vivona et al., 2010). When attached to GST, residues 680–728 of δ-adaptin could indeed be shown to bind to the VAMP7 longin domain as robustly as did GST δ-adaptin 650–797 (Figure 1D). Isothermal titration microcalorimetry showed the interaction to have a K_D_ of 14 ± 2 μM (Figure 1E), which is typical of clathrin adaptor/cargo interactions (Pryor et al., 2008), i.e., comparatively weak and readily reversible to allow subsequent cargo:adaptor dissociation. It is also in line with the weak interaction detected between VAMP7 and recombinant AP3 in pull-down experiments and between endogenous VAMP7 and AP3 in immunoprecipitation experiments (Figure 1B and see later).

### Structure of the VAMP7 Longin Domain:δ-Adaptin Hinge Complex

**Figure 2:**
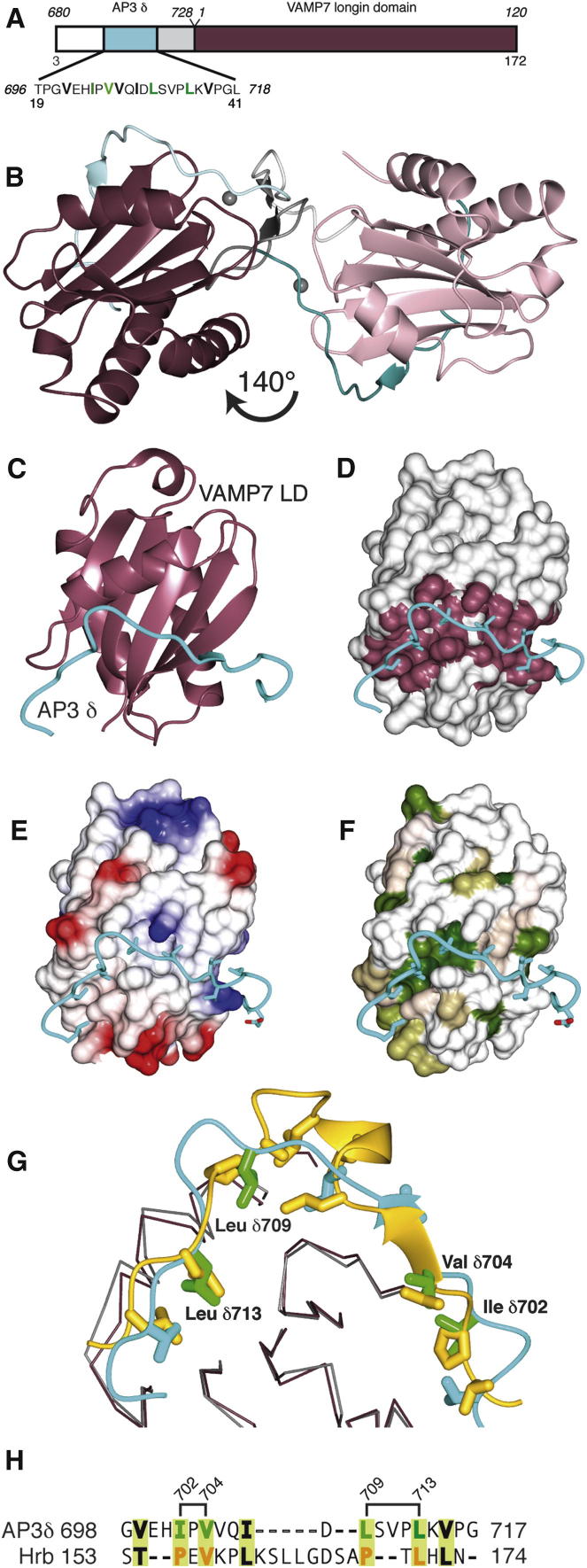
Structure of the δ-Adaptin Linker:VAMP7 Longin Domain Complex (A) Schematic representation of the δ-adaptin linker:VAMP7 longin domain fusion construct. As in all subsequent figures, the VAMP7 longin domain is shown in purple and the region of the δ-adaptin linker visible in the structure in cyan. The sequence of the δ-adaptin fragment is shown underneath. The numbers in italics correspond to the actual residue number in δ-adaptin, whereas those in regular font correspond to the numbers of the residues as they occur in the construct. (B) Structure of the linker exchanged dimer in the crystal's asymmetric unit. (C) Path followed by a δ-adaptin linker on a longin domain. (D) As (C) but with the longin domain shown in a gray surface representation with residues contacting the δ-adaptin linker colored purple. (E and F) As (C) but with longin domain surface colored by electrostatic potential (E) and hydrophobicity (ramped from polar (white) to hydrophobic (green)) (F) showing that the δ-adaptin linker sits in a hydrophobic groove. (G) Superposition of VAMP7:δ-adaptin linker (purple and cyan) and VAMP7:Hrb (gray and gold). Residues in Hrb (gold) spatially equivalent to those in δ-adaptin (green) mutation of which abolish the δ-adaptin/VAMP7 longin domain interaction are highlighted. (H) Structure based alignment of the δ-adaptin and Hrb fragments that bind the VAMP7 longin domain. Residues in bold mediate the interactions. Residues mutated in this studied are numbered. See also Figure S2.

In order to understand the molecular nature of the interaction through structural characterization, the δ-adaptin residues 680–728 were fused to the amino terminus of the longin domain (residues 1–120 Figure 2A). Crystals of this construct showed anisotropic diffraction to 2.8 Å in the best direction. The structure of the complex was readily solved by molecular replacement using as the search model the longin domain only of VAMP7 extracted from the Hrb/VAMP7 longin domain structure (Pryor et al., 2008). This showed two molecules in the asymmetric unit (Figure S2). Extra density could be seen for the δ-adaptin peptide (Figure S2). Automated model building followed by refinement allowed the whole complex (apart from the first 18 residues) to be built and refined to final R/Rfree of 0.24/0.30 (see Table 1 for structure determination statistics). The N-terminal region of the δ-adaptin linker (residues 20–40 of the construct, i.e., 696–718 of δ-adaptin) binds to the VAMP7 longin domain of the other molecule in the asymmetric unit, with the rest of the δ-adaptin peptide (residues 41–53 of the construct) linking the two longin domains via a small unsymmetrical β sheet (colored gray in Figure 2): neither this connecting region nor the two praseodymium ions of crystallization are involved in the VAMP7/δ-adaptin interaction. The two copies in the asymmetric unit, which represent the VAMP7 longin domain/δ-adaptin complex (i.e., excluding the connection arising from the fusion protein) are very similar in structure despite their different crystal environments, showing that the structure is not perturbed in the fusion protein construct.

### Mutating Residues in the VAMP7 Longin Domain:δ-Adaptin Hinge Interface Abolishes the Interaction In Vitro

**Figure 3:**
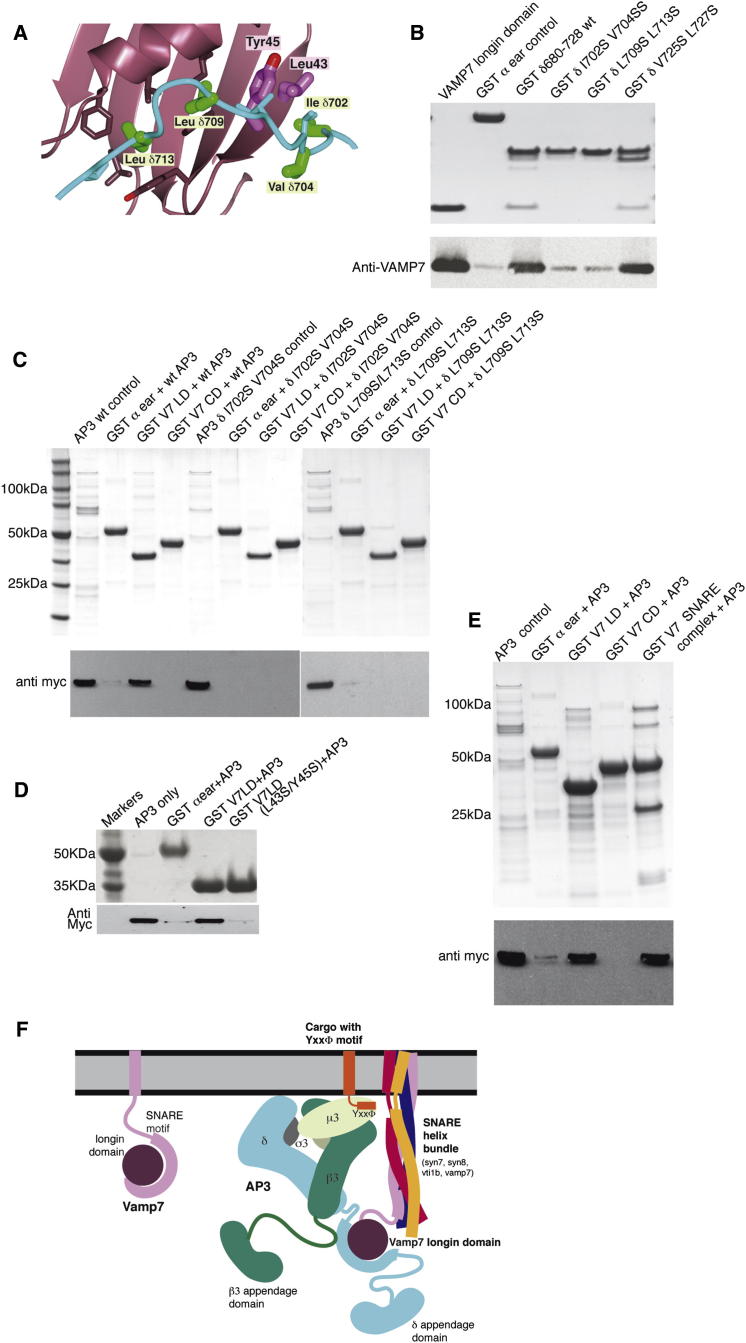
Molecular Mechanism of the Interaction between the δ-Adaptin Linker and VAMP7 Longin Domain (A) View into the δ-adaptin (cyan) binding slot on the VAMP7 longin domain (purple). Residues whose mutation abolish the δ-adaptin/VAMP longin domain interaction are colored green (δ-adaptin) and magenta (VAMP7 longin domain). (B) In the context of GSTδ-adaptin hinge (residues 680–728), mutations L709S/L713S and I702S/V704S reduced binding to VAMP longin domain in pull-down experiments by 95% and 92% respectively as compared with binding to GSTδ-adaptin wt (680–728), and GSTδ-adaptin (680–728)V725S/M727S. Top panel Coomassie blue staining and bottom panel western blot with anti-VAMP7 antibody. Blot quantified with ImageJ software with amount of binding to the negative control (GSTα-ear) being subtracted from all lanes. (C) In the context of recombinant AP3 containing full-length δ-adaptin, wt δ-adaptin binds to GST VAMP7 longin domain in GST pull-down experiments whereas mutations L709S/L713S and I702S/V704S in δ-adaptin abolished the interaction. Top panel Coomassie blue staining and bottom panel western blot with anti-Myc antibody. (D) GST VAMP7 longin domain (LD) but not mutant GST VAMP7 (L43S/Y45S) or GSTαear bind to AP3 containing full-length δ-adaptin and Myc-tagged μ3 subunit. Top panel Coomassie blue staining and bottom panel western blot with anti-Myc antibody. Lane 2 is loaded with AP3 only. (E) GST VAMP7 longin domain (LD) but not GST VAMP7 cytosolic domain (CD) or GSTαear bind to AP3 containing Myc-tagged μ3 subunit. Formation of an endosomal SNARE complex on VAMP7CD confers on VAMP7CD the ability to bind AP3. Top panel Coomassie blue staining and bottom panel western blot with anti-Myc antibody. Lane 2 is loaded with AP3 only. (F) Schematic representation of the binding of a *cis*-SNARE complex containing VAMP7 by AP3 through the VAMP7's longin domain colored as in (A). See also Figure S3.

Surprisingly, since there is no detectable sequence homology between the relevant portions of Hrb and δ-adaptin (Figure 2), the fragment of δ-adaptin binds to the longin domain following approximately the same route as that taken by Hrb (Pryor et al., 2008) and by VAMP7's own SNARE motif (as inferred from biochemical [Pryor et al., 2008] and NMR shift mapping [Vivona et al., 2010]). This δ-adaptin/Hrb binding site is a hydrophobic trough that runs half way round the VAMP7 longin domain (Figures 2C–2F). The interaction buries a total of around 1,990 Å^2^ of solvent accessible surface area (determined with PISA; Krissinel and Henrick, 2007) and is mediated by mainly hydrophobic residues (Figure 2G), which is typical of the low μM K_D_ interactions seen in the protein:protein interactions involved in transport vesicle formation. Mutation to serine of residues Ile702 and Val704 (mut1) and of Leu709 and Leu713 (mut2) in the δ-adaptin, which play key roles in the VAMP7:δ-adaptin interface (Figures 2H and 3A), reduced the interaction by at least 90% when introduced into the context of both the GSTδ-adaptin (680–728) linker and full-length AP3 as assessed by pull-downs (Figure 3) and isothermal titration microcalorimetry (linker only Figure S3), without affecting fold (as judged by circular dichroism and expression levels). That introduction of specific point mutations into recombinant AP3 abolishes VAMP7 longin domain binding demonstrates that only one binding site for VAMP7 exists on AP3 (confirmed by native immunoprecipitations see later). Mutation of residues that do not participate in the interface (Val725Ser/Leu727Ser) had no effect. The corresponding interface on the VAMP7 longin domain includes Leu4, Phe25, Val28, Ile32, Lys42, Leu43, Tyr45, Tyr50, and Phe52. GST pull-down experiments and isothermal titration microcalorimetry (Figure 3; Figure S3) showed that mutation of key interface residues Leu43Ser/Tyr45Ser on VAMP7 longin domain abolished its interaction with δ-adaptin without affecting the fold of the longin domain as determined by CD (data not shown): this mutation also abolished the interaction between the VAMP7 longin domain and Hrb (Pryor et al., 2008).

### VAMP7 Can Only Bind δ-Adaptin When Its SNARE Motif Is Involved in SNARE Complex Formation

Since δ-adaptin and Hrb bind to the same site on the VAMP7 longin domain onto which VAMP7's own SNARE motif can also bind back (Pryor et al., 2008), the binding of the δ-adaptin to the VAMP7 longin domain would be expected to be mutually exclusive with the SNARE motif binding back onto the longin domain. This is indeed the case since no binding was seen between the full cytosolic domain of VAMP7 and AP3 either by GST pull-downs or in our Y2H analysis. The inhibition of VAMP7:δ-adaptin binding by the intramolecular binding back of the SNARE motif can, however, be relieved by the removal of the SNARE motif from its binding site on the longin domain through its participation in SNARE complex formation with its cognate endosomal SNAREs syntaxin7, syntaxin8, and vti1b (Figure 3) or cognate plasma membrane SNAREs (data not shown) implying that when trafficked by AP3, VAMP7 must be part of a *cis*-SNARE complex.

### The VAMP7 Longin Domain: δ-Adaptin Interaction Occurs In Vivo but Is Not Required for the Recruitment of AP3 to Membranes

**Figure 4:**
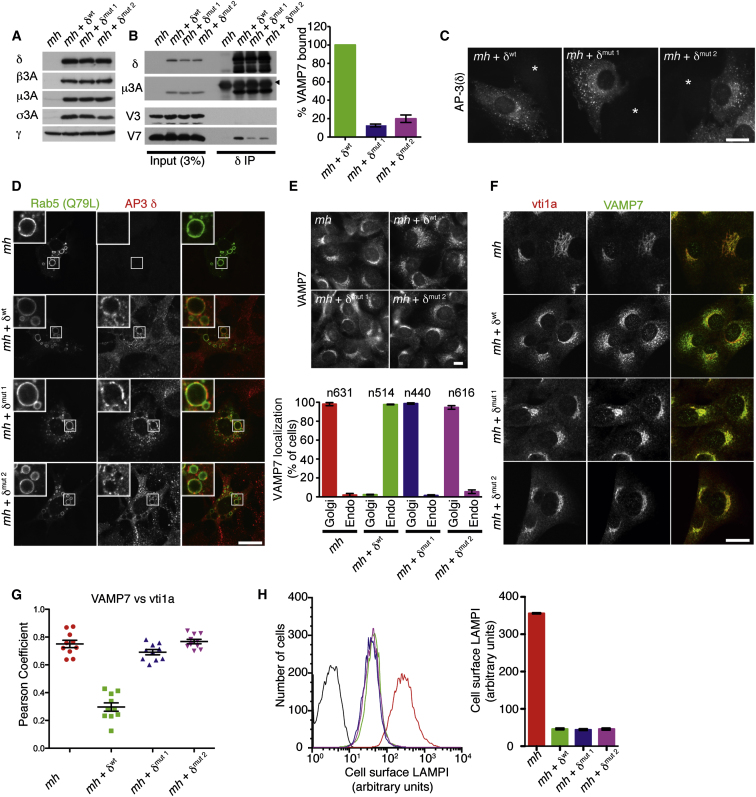
The Interaction between VAMP7 and AP3 Is Required for VAMP7's Steady-State Localization but Not Lysosomal Protein Targeting (A) *Mocha* fibroblasts were retrovirally transduced with δ^wt^, δ^mut1^, and δ^mut2^ adaptin and levels of the AP3 subunits determined by immunoblotting. γ-Adaptin a subunit of the AP-1 adaptor complex and was used as a loading control. (B) *Mocha* fibroblasts were retrovirally transduced either with δ^wt^, δ^mut1^, and δ^mut2^ adaptin and native immunoprecipitations performed with antibodies to δ-adaptin. The immunoprecipitations were immunoblotted for components of the AP-3 complex (μ3A and δ) and the post-Golgi R-SNAREs VAMP3 (negative control) and VAMP7. Immunoprecipitations were performed 3 times and the level of VAMP7 bound to AP-3 quantified (see graph). Error bars show the SEM. (C) *Mocha* fibroblasts were mixed with cells either expressing δ^wt^, δ^mut1^, and δ^mut2^ adaptin and then fixed and stained with antibodies to δ-adaptin. Asterisk indicates nontransduced cell. Scale bar, 20 μm. (D) *Mocha* fibroblasts expressing δ^wt^, δ^mut1^, and δ^mut2^ were transfected with GTP locked GFP-Rab5A. The cells were then fixed and stained with antibodies against δ adaptin. Scale bar 20 μm. (E) *Mocha* fibroblasts expressing δ^wt^, δ^mut1^, and δ^mut2^ adaptin were fixed and stained with antibodies VAMP7. The micrographs show representative fields of cells. To quantify the localization of VAMP7, over 10, random micrographs were taken for each cell population and the localization of VAMP7 scored manually. The experiment was repeated 3 times and the error bars show the SEM n = total number of cells scored in the three independent experiments. Scale bar, 10 μm. (F) *Mocha* fibroblasts expressing δ^wt^, δ^mut1^, and δ^mut2^ were fixed and stained with antibodies to VAMP7 and vti1a. Scale bar 20 μm. (G) The level of colocalization between VAMP7 and vti1a was calculated using Volocity software. The bar graphs show mean Pearson coefficients and the error bars SEM 10 cells were imaged for each condition. (H) *Mocha* fibroblasts reterovirally transduced either with δ^wt^, δ^mut1^, and δ^mut2^ adaptin and the cell surface levels of LAMP1 determined using flow cytometry. The raw data from a representative experiment is shown in the histogram (black line unstained-control, red-*mh*, green-*mh* + δ^wt^, blue-*mh* + δ^mut1^ and magenta-*mh* + δ^mut2^). The bar graph show the data generated from three independent experiments. Error bars show experimental range. See also Figures S4 and S5.

*Mocha* cells were transduced with either wild-type (δ^wt^) or VAMP7 nonbinding mutants Ile702Ser/Val704Ser (δ^mut1^) or Leu709Ser/Leu713Ser (δ^mut2^) δ-adaptin. Re-expression of wt and mutant δ-adaptin led to similar levels of stabilization of the other subunits of the complex (Figure 4A) indicating that the mutations did not affect complex assembly and stability.

To determine whether these mutations abolish VAMP7 binding to AP3 in vivo, native immunoprecipitations were performed from *mocha* cells expressing δ^wt^, δ^mut1^, or δ^mut2^ with antibodies against the δ subunit of the AP3 complex. The immunoprecipitations were blotted for components of the AP3 complex and the post-Golgi R-SNAREs VAMP3 and VAMP7 (Figure 4B). VAMP7 was coimmunoprecipitated with wt AP3. However, the levels of VAMP7 coimmunoprecipitated with the mutant AP3 complexes were reduced by more than 80%. In order to determine whether the inhibition of VAMP7 binding to AP3 altered AP3 recruitment to membranes, *mocha* cells expressing δ^wt^, δ^mut1^, or δ^mut2^ were stained with antibodies to δ adaptin. The δ^mut^ constructs localize to punctate, membrane associated structures that are indistinguishable from those on which the δ^wt^ was found indicating that inhibiting VAMP7 binding does not affect AP3 recruitment on to membranes (Figure 4C) presumably because AP3's major determinant of recruitment is binding to membrane-associated Arf1:GTP (Ooi et al., 1998). To investigate further whether the targeting of AP3 complexes that cannot bind VAMP7 to early endosomes is altered we have colocalized AP3 with Rab5 (Figure 4D). It has previously been shown that AP3 is found in discrete sorting microdomains on the limiting membrane of Rab5 positive swollen endosomes when Rab5a (Q79L) is overexpressed (Craige et al., 2008). *Mocha* cells expressing δ^wt^, δ^mut1^, or δ^mut2^ were transiently transfected with GFP-Rab5 (Q79L) and stained for δ adaptin. As expected, wt AP3 was associated with these structures. The mutant forms of the AP3 were also found on these structures indicating that the targeting of AP3 complexes to Rab5 positive membranes does not require VAMP7 binding. In support of the finding that correct AP3 targeting is independent of VAMP7 binding we observed that LAMPI trafficking, which would require correct membrane localization of AP3 to early/recycling endosomes is rescued equally well by the wt and mutant AP3 complexes (see Figure 4H).

### AP3 Traffics VAMP7 from Early/Recycling Compartments of the Endocytic Pathway to Late Endosomes/Lysosomes

To determine whether the interaction between AP3 and VAMP7 is required for endogenous VAMP7's steady-state localization, *mocha* fibroblasts transduced with δ^wt^, δ^mut1^, or δ^mut2^ were stained with antibodies to VAMP7 (Figure 4E). In cells expressing wild-type AP3, VAMP7 was, as expected, predominantly localized to punctate structures that show some colocalization with the TGN markers vti1A (Figure 4F) and TGN38 (Figure S4) and the endosomal/lysosomal markers TF-R (data not shown), Rab7 (data not shown), and LAMPI (Figure S4). In untransduced *mocha* cells, or those expressing δ^mut1^ or δ^mut2^, VAMP7's localization is altered so that it predominantly localizes to a reticular, perinuclear compartment that colocalizes with the TGN markers vti1A (Figure 4F) and TGN38 (Figure S4). In addition, in *pearl* fibroblasts, which lack AP3 complexes due to the loss of β3A, VAMP7 also accumulates in the TGN (data not shown) indicating that this phenotype is not cell line specific but is a general consequence of a loss of AP3. Together these data demonstrate that the trafficking of endogenous VAMP7 is dependent on its binding to AP3.

AP3 is thought to function on endosomes (Peden et al., 2004; Theos et al., 2005) so it is counterintuitive that VAMP7 is accumulating at the TGN when AP3 is lost from cells or when the interaction between AP3 and VAMP7 is abolished. One possible explanation for this phenotype is that failure to sort VAMP7 from endosomes results in VAMP7 being recycled back to the TGN. To test this hypothesis, we have perturbed the recycling pathway using the drug chloroquine. Chloroquine inhibits the transport of protein between endosomes and the TGN resulting in the accumulation of TGN localized proteins in LAMPI positive endosomes (Chapman and Munro, 1994; Reaves et al., 1998). Mocha fibroblasts expressing δ^wt^, δ^mut1^, and δ^mut2^ were treated with 50 μM chloroquine for 2 hr and fixed then stained for LAMPI and VAMPs 4 and 7. VAMP4 is a TGN localized R-SNARE that actively cycles between endosomes and the TGN (Tran et al., 2007). As expected, VAMP4 became mislocalized to LAMPI positive endosomes in the presence of chloroquine in all three cell types (Figure S5, arrows indicate areas of colocalization). Treatment of untransduced *mocha* cells or *mocha* cells expressing δ^mut1^ or δ^mut2^ with chloroquine resulted in a redistribution of a significant fraction of VAMP7 from the TGN to LAMPI positive endosomes (Figure S4), arrows indicate areas of colocalization). A similar redistribution was also observed for VAMP7 in *pearl* cells (data not shown). These data point to VAMP7 being able to cycle between endosomes and the TGN, such that it accumulates in the TGN in the absence of AP3 or the presence of AP3 mutants to which it cannot bind. Consistent with this model, it should be noted that in a previous immunoelectron microscopic study on PC12 cells, about 30% of VAMP7 was localized in or close to the TGN (Advani et al., 1998). Finally, in order to investigate whether the VAMP7 AP3 interaction is required for the correct trafficking of lysosomal membrane proteins, the cell surface levels of LAMPI in *mocha* cells transduced with δ^wt^, δ^mut1^, or δ^mut2^ were measured (Figure 4H). As previously shown (Peden et al., 2004), re-expression of δ^wt^ in *mocha* cells significantly decreased the cell surface levels of LAMPI by redirecting its trafficking. Interestingly, δ^mut1&2^ were similarly capable of reducing the cell surface levels of LAMPI, indicating, that the AP3/VAMP7 interaction is not required for the correct sorting of LAMP1.

## Discussion

The interaction between VAMP7 and the linker region of δ-adaptin from the AP3 adaptor complex is another example of post-Golgi SNARE trafficking mediated through a highly specific, μM K_D_ interaction of the SNARE with a coat component that will not compete with standard cargo selection. However, rather than use a clathrin adaptor with no other known cargo selective function, in this case VAMP7 uses a different site on a cargo adaptor that binds standard YxxΦ/ExxxLL motif containing cargo elsewhere and thus defines yet another mode of cargo binding by members of the heterotetrameric clathrin adaptor family. Intriguingly, in *Saccharomyces cerevisiae* the VAMP7 homolog (Nyv1p) is also trafficked to a late endocytic compartment (the vacuole) by AP3, but the molecular mechanism is not conserved, as in yeast a YxxΦ motif in a surface loop of Nyv1p binds to the μ3 subunit (Wen et al., 2006). As in the case of the endocytosis of VAMP7 by Hrb, in order to bind to its cognate vesicle coat partner δ-adaptin, VAMP7 must be part of a *cis*-SNARE complex. In respect of this, it is interesting to note that in yeast AP3 has been proposed to be present on carrier vesicles that also contain the SNARE Vam3p, the homolog of mammalian Syntaxin7, a SNARE with which we expect VAMP7 to form complexes (Darsow et al., 1998).

The sequential binding and trafficking of VAMP7 by first Hrb at the plasma membrane for movement to an early/recycling endosome and then by AP3 from such an endosome to late endosome/lysosomal compartment provides a simple route by which VAMP7 can be moved back down the endocytic pathway to its steady-state location following fusion of endosomes/lysosomes with the plasma membrane, for example during mitosis (Boucrot and Kirchhausen, 2007) membrane repair (Rao et al., 2004) and neurite outgrowth (Coco et al., 1999) while maintaining it in an inactive form and so avoiding the danger of causing unwanted membrane fusion events. It also provides a route by which VAMP7 and its SNARE partners can be moved after their biosynthesis via an early/recycling endosome to a late endosome/lysosome. By binding to the δ-adaptin subunit of AP3, VAMP7 is also implicated in the generation of lysosome related organelles such as the melanosome since AP3 has been shown to play a critical role in their formation (Theos et al., 2005). The identification of a point mutant version of AP3 that in all other respects is fully functional may allow for VAMP7's role in these biogenesis processes to be studied in vivo. Finally, since abolition of VAMP7 binding does not inhibit AP3-mediated delivery of other proteins to late endosomal compartments and also in view of the deduction that VAMP7 is transported as part of an inactive *cis*-SNARE complex, it seems likely that VAMP7 is not responsible for the membrane fusion event needed to complete this delivery step. A different R-SNARE, most likely VAMP8 (Antonin et al., 2000; Pryor et al., 2004) must catalyze the necessary fusion event and the VAMP7 is transported to late endocytic compartments so that it can used in subsequent lysosomal functions.

## Experimental Procedures

For constructs and antibodies used, see Supplemental Information.

### Protein Expression and Purification

All individual recombinant proteins were expressed in Rosetta2(DE3)pLysS cells for 16–20 hr at 22°C following induction with 0.2 mM IPTG at 37°C. Cells were lysed using an EmulsiFlex continuous flow French press in 20 mM HEPES (pH 7.4), 200 mM NaCl (buffer A), 1 mM DTT, and protease inhibitors and the proteins affinity purified on glutathione-Sepharose or Ni-NTA-agarose as appropriate. Following affinity purification all proteins were subsequently purified by Superdex 75 gel filtration with the exception of AP3 that was purified on Superdex S200 gel filtration.

### Reconstitution of SNARE Complexes

All SNARE purifications and SNARE complex reconstitution were performed in buffer B (20 mM HEPES [pH 7.4], 500 mM NaCl, 5 mM DTT) at 4°C. Syntaxin7, Syntaxin8, and Vti1b were purified on glutathione-Sepharose and the GST subsequently cleaved by overnight incubation of the beads with Prescission protease. The cleaved SNAREs were eluted from the glutathione-Sepharose and subsequently purified by S75 gel filtration. GSTVAMP7 was purified as described above. The purified SNAREs were mixed in an approximate molar ratio of syntaxin7:syntaxin8:Vti1b:GSTVAMP7 of 4:4:4:1 and incubated for 16 hr with constant agitation. SNARE complexes were isolated on glutathione-Sepharose and after their elution subsequently purified by Superdex S200 gel filtration column.

### Protein:Protein Interaction Assays

All assays were performed using GST tagged protein as the “bait.” Assays involving AP3 complexes were performed in buffer A plus 2 mM DTT and 2 mM ß-mercaptoethanol and 0.5% NP40. All other assays were performed in buffer A plus 2 mM DTT and 2 mM ß-mercaptoethanol and 0.5% NP40.

Equimolar amounts (1 nmol) of GST fused bait was bound to 80 μl of a 50% slurry of glutathione Sepharose. An approximate 5-fold excess of potential binding proteins (“prey”) (i.e., approximately 5 nmol) was added and the volume made up to 1 ml with buffer. These were mixed at 4°C for 1 hr, then washed three times for 5 min in 1 ml buffer A. Bound proteins were analyzed by Coomassie brilliant blue staining and western blotting.

### Isothermal Titration Calorimetry Experiments

All experiments were performed using a Microcal Auto-iTC200. Proteins were exchanged into 50 mM HEPES [pH 7.4], 200 mM NaCl, 4 mM ß-mercaptoethanol on Superdex75 gel filtration. VAMP7 longin domain (0.3 ml) was placed in the cell at 0.15–0.2 mM, and GST delta constructs at 0.8–1.2 mM were titrated in 20–30 injections of 2μl. Titration curves were tfitted using ORIGIN software (http://www.originlab.com/). All experiments were carried out three to five times and SD values are shown.

### Purification and Crystallization and Structure Determination

GSTVAMP7(1-120):δ−adaptin(680–728) was first purified on glutathione-Sepharose followed by Superdex75 gel filtration. The GST was cleaved by thrombin digestion at 4°C overnight, which left an additional two residues at the N-terminus of the VAMP7 longin domain. Cleaved GST was subsequently removed by passing the cleavage products back down glutathione-Sepharose. The resulting VAMP7(1-120):δ−adaptin(680–728) was again purified by Superdex 75 gel filtration and concentrated to 12 mg/ml. The best crystals were grown against a reservoir containing 4M NaCl, 100 mM bicine (pH 9.0), 10 mM praseodymium acetate over a period of 4–6 weeks. Diffraction was severely anisotropic, with spots extending to around 2.8 Å resolution along c, but only to about 3.4 Å resolution along a or b. The structure was solved by molecular replacement searches with the VAMP7 longin domain from the VAMP7/Hrb complex (PDB code 2vx8). For a detailed description of the structure determination see Supplemental Information. Coordinates and structure factors have been deposited in the PDB with accession code 4afi.

### Flow Cytometry and Cell Biology

*Mocha* fibroblasts (CRL-2709; American Type Culture Collection) were retrovirally transduced with either wild-type or mutant, Ile702Ser/Val704Ser (mut 1) and Leu709Ser/Leu713Ser (mut 2) δ−adaptin, as previously described (Peden et al., 2004). Immunoblotting and immunoprecipitations were performed as in (Gordon et al., 2010). The cell surface level of LAMPI in the transduced fibroblasts was quantified as previously described (Gordon et al., 2009). One microgram of anti-LAMPI-Alexa647 was used to stain approximately 100,000 cells. The samples were analyzed on a BD FACSCalibur (approximately 10,000 cells were gated for forward/side scatter and 7AAD exclusion). The mean fluorescence for each sample was then calculated using the software FlowJo (Tree Star, Inc., Ashland, OR). Transduced cells were fixed, stained and the images acquired and processed as in (Gordon et al., 2010). The level of colocalization between VAMP7 and Vti1A and LAMPI was determined using Volocity (Perkin Elmer). To quantify the levels of VAMP7 coimmunoprecipitated with AP3, autoradiographs generated from the immunoblots were scanned and the signal intensity of the bands calculated using the ImageJ.

## Figures and Tables

**Table 1:** Statistics for Crystallographic Structure Determination

Data Processing		
Data set number (wavelength Å)	1, 0.9393	2, 1.500
Resolution range (Å)	61–2.80 (2.95–2.80)	61–2.80 (2.95–2.80)
Unit cell lengths a, c (Å)	63.6, 217.9	63.0, 218.0
Beamlines	Diamond I03	Diamond I03
Number of crystals	2	1
R_merge_	0.306 (2.9)	0.196 (3.3)
R_merge_ in top intensity bin	0.073	0.049
R_meas_	0.329 (3.1)	0.214 (3.6)
R_pim_	0.090 (0.83)	0.067 (1.0)
Number of reflections	11,807	11,583
Mean ([I]/sd[I])	7.7 (1.2)	8.6 (1.0)
Half-dataset correlation coefficient CC_1/2_	0.995 (0.62)	0.997 (0.65)
Resolution limit along a^∗^, c^∗^ (Å)^a^	3.37, 2.8 (3.41, 2.8)	3.36, 2.8 (3.69, 2.8)
Completeness (%)	100.0 (100.0)	99.9 (99.6)
Multiplicity	13.1 (13.6)	11.0 (11.6)
Anomalous completeness (%)	100.0 (100.0)	99.9 (99.6)
Anomalous multiplicity	7.3 (7.3)	6.2 (6.2)
Δ_anom_ Correlation between half-sets^b^	0.016 (inner 0.39)	0.419 (inner 0.823)
Wilson plot B (Å^2^)	63	79

**Refinement**		

R-factor (3.0–2.8 Å)	0.24 (0.40)	0.27 (0.44)
R_free_ (3.0–2.8 Å)	0.26 (0.43)	0.29 (0.43)
Number of reflections (number R_free_)	11,194 (551)	10,999 (544)
Number of atoms (protein, Pr^3+^, H_2_O)	2,438, 2, 4	2,438, 2, 4
<B > (Å^2^)	58	61
Anisotropic B (B11, B33)	3.14, −6.29	3.73, −7.47
f” for Pr^3+^ ions (e, theoretical)	4.56	9.86
Rms bond length deviation (Å)	0.011	0.011
Rms angle deviation (°)	1.5	1.6
Rms ΔB (bonds, angles)	7.6, 10.2	7.5, 10.0
Ramachandran outliers	1%	1%
Ramachandran favored	94.7%	94.4%

R_merge_ = Σ(I_hl_ - < I_h_ >)/Σ < I_h_ >. R_meas_ = Σ√(n_h_/n_h_-1)(I_hl_ - < I_h_ >)/Σ < I_h_ >. R_pim_ = Σ√(1/n_h_-1)(I_hl_ - < I_h_ >)/Σ < I_h_ >.

a Resolution limit estimated in cones of half-angle 20° around a^∗^ (= b^∗^) and c^∗^ axes, from the point at which the half-dataset correlation coefficient falls below 0.5 (or < < I > /sd < I > > falls below 2.0).

b “Inner” resolution range ∞ to 8.8 Å.

## Supplemental Information

Document S1. Figures S1–S5 and Supplemental Experimental Procedures
