# Supplementary material for: Structural Basis of the Intracellular Sorting of the SNARE VAMP7 by the AP3 Adaptor Complex
Source: Dev Cell. 2012 May 15;22(5):979–88. doi: 10.1016/j.devcel.2012.01.018 (PMC3549491; doi:10.1016/j.devcel.2012.01.018)
Supplement: Document S1. Figures S1–S5 and Supplemental Experimental Procedures [file mmc1.pdf]

**Developmental Cell, Volume *22***

## Supplemental Information

# Structural Basis of the Intracellular Sorting of the SNARE VAMP7 by the AP3 Adaptor Complex

**Helen M. Kent, Philip R. Evans, Ingmar B. Schäfer, Sally R. Gray, Christopher M. Sanderson, J. Paul Luzio, Andrew A. Peden, and David J. Owen**

## Inventory of Supplemental Information

## Supplemental Figures and Legends

Supplementary Figure S1 linked to Figure 1

Supplementary Figure S2 linked to Figure 2

Supplementary Figure S3 linked to Figure 3

Supplementary Figure S4 linked to Figure 4

Supplementary Figure S5 linked to Figure 4

## Supplemental Information

Constructs, antibodies, and full structure determination methods

## Supplemental References

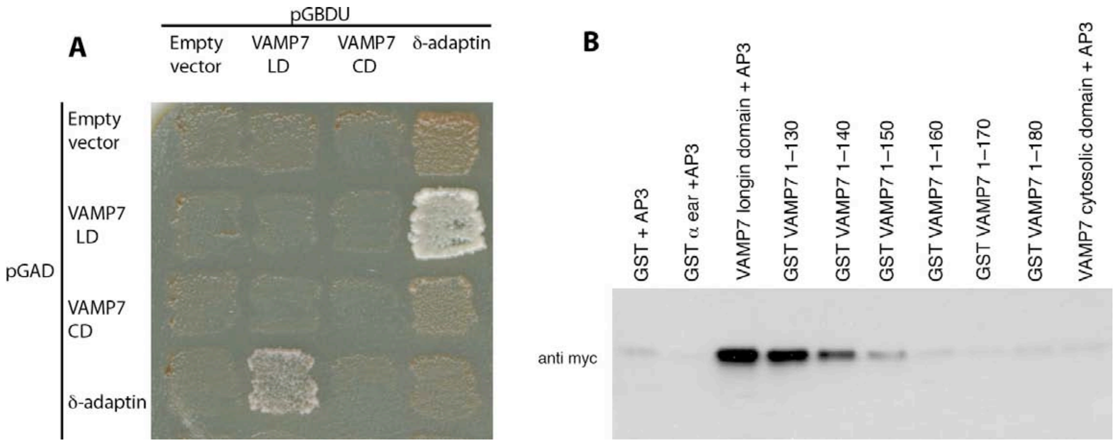


**Figure S1 associated with figure 1**. **A**. Y2H interaction analysis showing that after 4 days at 30°C interactions between the VAMP7 longin domain (LD) and δ-adaptin but not between full length VAMP7 cytoplasmic domain (CD) and δ-adaptin could be detected. No autoactivation was detected. **B**. GST pull down experiments using sequential truncations of the unstructured SNARE motif of VAMP7. Only constructs comprising residues 1-120 (the longin domain), 1-130 and 1-140 bound to AP3 containing full length δ-adaptin and Myc-tagged μ3A adaptin. Western blot of SDS PAGE developed with antiMyc antibody 9E10.

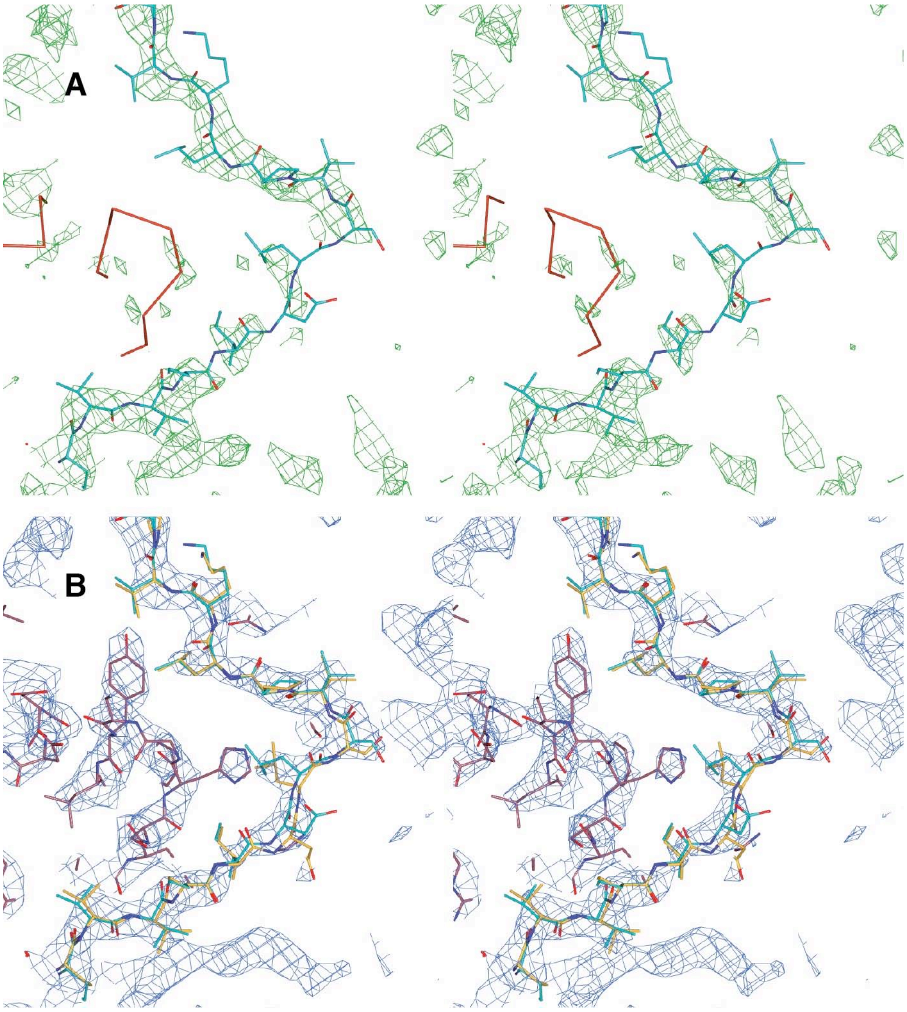


**Figure S2 associated with figure 2**. **A.** Stereo view of difference electron density for the δ-adaptin binding region after molecular replacement with the VAMP7 longin domain, contoured at 0. 29e/Å, with the final refined model superimposed (cyan). The map was calculated from a rigid body refinement in Refmac with the SAD target against dataset 1, using anomalous scattering from the two $Pr^{3+}$ ions. **B**. Map and model (gold) from automatic model building with Buccaneer for the δ-adaptin binding region, contoured at 0.29e/Å, with the final refined model

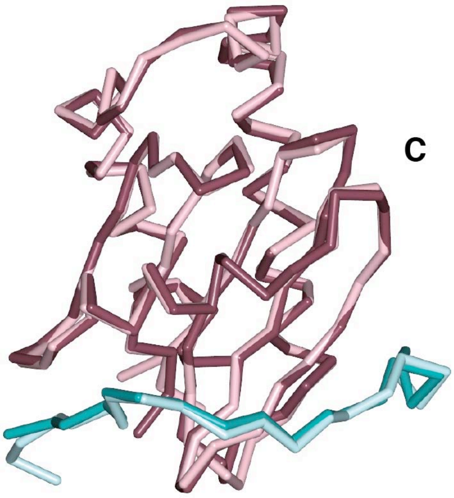

superimposed (cyan) **C**. Superposition of Cα traces of the two VAMP7 longin domains in the asymmetric unit (pink and purple) along with their δ-adaptin binding regions, showing that the two molecules are very similar despite their asymmetric assembly.

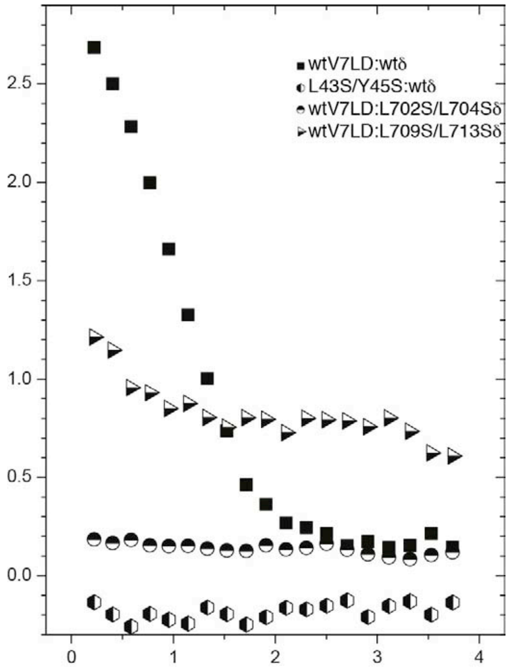


**Figure S3 associated with figure 3.** Mutation in critical residues of the VAMP7: δ-adaptin interface either in the VAMP7 longin domain (L43S/Y45S) or in the unstructured δ-adaptin hinge (I702S/V704S) or (L709S/L713S) reduced binding below levels detectable by itc $K_D$ >300μM. The binding of the two wt proteins ($K_D$ 14μM) is shown for comparison

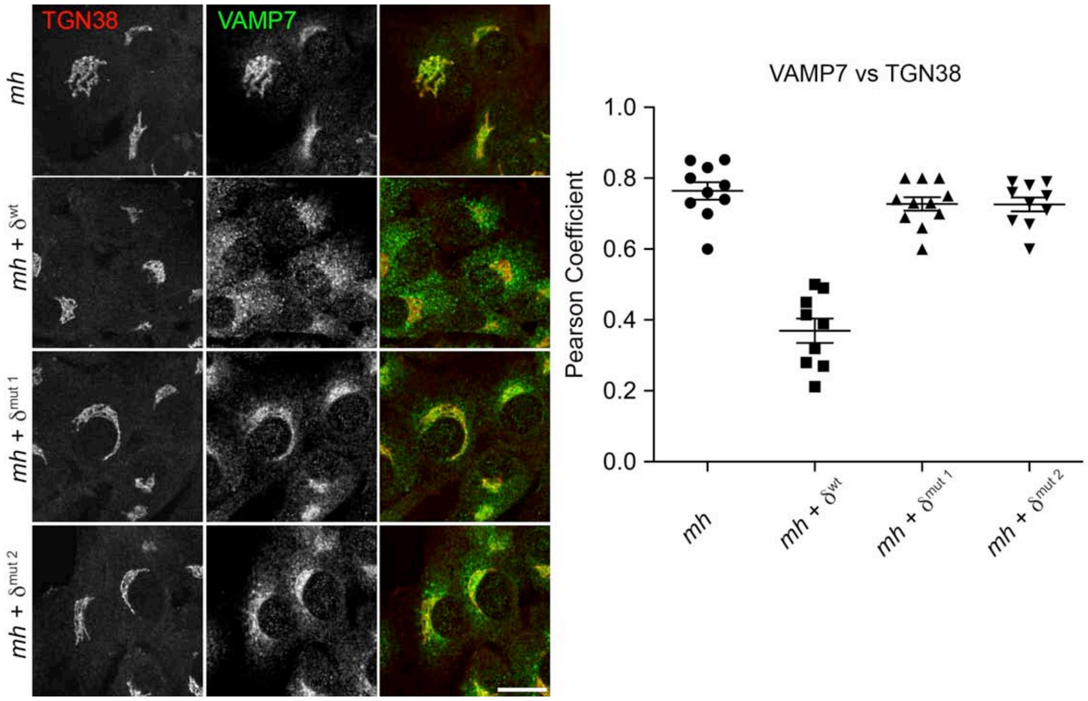


**Figure S4 associated with Figure 4.** Endogenous VAMP7 is localized to the TGN in cells lacking AP3 or AP3 mutant versions of AP3 that can no longer bind VAMP7. Mocha fibroblasts expressing δwt, δmut1 (I702S/V704S), or

δmut2 (L709S/L713S) were fixed and stained with antibodies to VAMP7 and TGN38. Scale bar 20µm. The level of colocalisation between VAMP7 and TGN38 was calculated using Volocity software. The bar graphs show mean Pearson coefficients and the error bars SEM. 10 cells were imaged for each condition.

**Figure S5 associated with Figure 4**. VAMP7 is cycling between the TGN and endosomes in cells lacking AP-3 or AP-3 mutants that can no longer bind VAMP7 Mocha fibroblasts expressing δwt, δmut1 (I702S/V704S), or δmut2 (L709S/L713S) were incubated with 50µM chloroquine for 2 hours at 37°C and then fixed and stained with antibodies against VAMPs 4, 7 and LAMP1. Scale bar 20µm. The level of colocalisation between VAMP7 and LAMPI (C) was calculated using Volocity software. The bar graphs show mean Pearson coefficients and the error bars SEM. Between 5 and 10 cells were imaged for each condition.

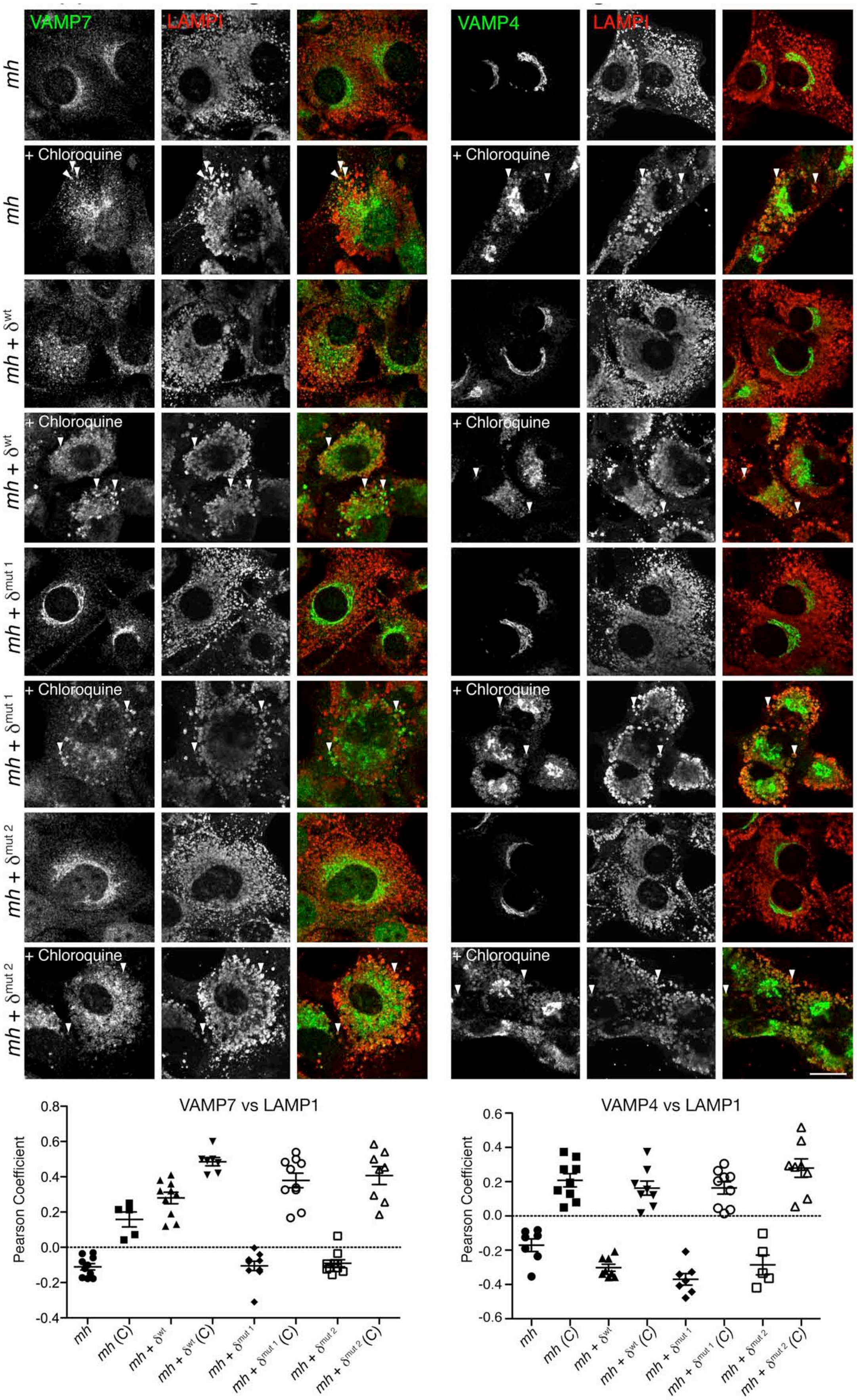

VAMP7
LAMP1
VAMP4
LAMP1
mh
+ Chloroquine
mh + δ^wt
mh + δ^mut 1
mh + δ^mut 2
VAMP7 vs LAMP1
VAMP4 vs LAMP1
Pearson Coefficient
mh
mh (C)
mh + δ^wt
mh + δ^wt (C)
mh + δ^mut 1
mh + δ^mut 1 (C)
mh + δ^mut 2
mh + δ^mut 2 (C)

**Constructs used**

pMWH6β3A(human)(1-650) + μ3Amyc(rat), pMWKan δ(Pan troglodytes)1-1203 + σ3(human), pMWKan δ(Pan troglodytes)1-797 + σ3(human)

pGEX4T2VAMP7(1-120), pGEX4T2VAMP7(1-188), pMWH6VAMP7 (1-120), pMWH6VAMP7 (1-120) (Leu43Ser/Tyr45Ser), pMWH6VAMP7 (1-188)

pGEX4T2 δ-adaptin(680-728):VAMP7(1-120)

pGEX6P1Syntaxin7(169-237), pGEX6P1Syntaxin8(149-213), pGEX6P1Vti1B(1-206)

pGEX4T2δ (650-797), pGEX4T2δ-adaptin (700-740), pGEX4T2δ-adaptin(700-740)Leu709Ser/Leu713Ser, pGEX4T2δ-adaptin (700-740)Leu718Ser/Met720Ser, pGEX4T2δ-adaptin (700-740)Val725Ser/Leu727Ser)

pBMN δ-adaptin (1-1203), pBMN δ-adaptin (1-1203) Ile702Ser/Val704Ser, pBMN δ-adaptin (1-1203) Leu709Ser/Leu713Ser.

pEGFP-C1-Rab5A (1-215) Gln79Leu the construct was generously provided by Dr Victor Faundez.

**Antibodies and Microscopy**

Mouse anti-δ adaptin (SA4), rabbit anti-σ3A, μ3A, β3A and γ-adaptin have previously been described (Peden et al., 2004; Simpson et al., 1997). Anti-VAMP7 longin-domain specific antibodies were generated by immunising rabbits with bacterially expressed GST-VAMP7 (mouse 1-120) and affinity purified as previously described (Steegmaier et al., 1999). The anti-LAMP1 antibody (1D4B) (Hughes and August, 1981) was obtained from the

Developmental Studies Hybridoma Bank developed under the auspices of the NICHD and maintained by The University of Iowa, Department of Biological Sciences, Iowa City, IA. The LAMP1 antibody was conjugated to Alexa-647 using a protein-labeling kit according to the manufacturer's instructions (Molecular Probes, Inc.). Transduced cells were fixed, stained and the images aquired and processed as in (Peden et al., 2004).

**Structure determination**

Crystals of δ-adaptin(680-729):VAMP7(1-120) belonged to space group $P4_32_12$, unit cell a = b = 63.3Å, c = 218.2Å. Diffraction measured at the Diamond Light Source synchrotron was severely anisotropic. Two datasets were used, dataset 1 collected at short wavelength (0.9393Å) for which data were combined from 2 crystals, and a second collected at 1.5Å wavelength to enhance the anomalous signal from the $Pr^{3+}$ ions of crystallisation (table 1): merging statistics are relatively poor due to the anisotropy. For set 1, the data merged from two crystals gave better scores than the individual crystals both in molecular replacement and in refinement. Images were integrated with Mosflm (Leslie, 2006) and scaled with Scala or Aimless (Evans, 2006). The data extended to 2.8Å resolution along c* but only to around 3.4Å perpendicular to c* (a*b* plane), as judged by half-dataset correlation coefficients ($CC_{1/2}$) for reflections in cones along the axes. Molecular replacement searches with the VAMP7 longin domain from the VAMP7/Hrb complex (PDB code 2vx8) using Phaser (McCoy et al., 2007) gave a clear solution for two molecules in the asymmetric unit (log-likelihood gain 555,

Z=24). A strong $Pr^{3+}$ ion was visible on each molecule, also shown in anomalous difference maps. For both datasets, difference maps calculated with Refmac, using the anomalous scattering from the $Pr^{3+}$ ions (SAD target), showed electron density in the groove on VAMP7 which was occupied by Hrb in its complex (Figure S1A). Automated model building with Buccaneer & Refmac (Cowtan, 2006) built additional residues into the grooves on both molecules: six model-building trials were run, against the two datasets and using different options, leading to twelve models for the δ-adaptin region: eight of these assigned the AP3 δ-adaptin sequence to the density consistently, mostly fitting the electron density clearly (fig S1B), the other four were inconsistently different and did not fit the density well as judged by residue-by-residue map correlation coefficients. The consensus was accepted as the starting model. The registration of the sequence was confirmed by manual building of a poly-Ala chain through the "peptide" region, and using the assign_sequence program from Phenix to give an unambiguous assignment in agreement with Buccaneer (Adams et al.). The structure was completed by manual model building with Coot (Emsley and Cowtan, 2004) and refinement with Refmac, using the SAD target, with local NCS restraints (Murshudov et al., 1997) and external distance restraints derived from the previously determined VAMP7 longin domain (2vx8) generated with the program ProSmart (Murshudov et al., 2011). Dataset 2 gave slightly clearer results in the automatic model building, but refinement statistics were better for dataset 1 (Table 1): there was no significant difference between models refined against the two datasets. Residues 18-40 bind in the VAMP7 groove in similar

ways in both molecules. Interpretation of the "linker" region 40-53 was more difficult since crystallographic and non-crystallographic symmetry bring several molecules close together, and it was hard to decide the way in which the molecules were connected. As refinement progressed however, it became clear that the linker forms a small asymmetric β-sheet between the longin domains, and that the two chains cross over so that the δ-adaptin region 697-719 binds to the other longin domain in the asymmetric unit (Figure 2). The two longin domains are related by a screw rotation of 140° with a 19.3Å translation, but despite the asymmetry of their packing and of the linker, the domains themselves, along with the bound δ-adaptin region, are very similar (Figure S1C), indicating that the structure of the Vamp7/δ-adaptin complex is not a crystal artifact due to the use of a fusion protein. The buried surface of the interaction between the δ-adaptin linker and VAMP7 longin domain was calculated as 940Å$^2$ using PISA (Krissinel and Henrick, 2007)

**Supplementary References**


Cowtan, K. (2006). The Buccaneer software for automated model building. 1. Tracing protein chains. Acta Crystallogr D Biol Crystallogr *62*, 1002-1011.

Emsley, P., and Cowtan, K. (2004). Coot: model-building tools for molecular graphics. Acta Crystallogr D Biol Crystallogr *60*, 2126-2132.

Evans, P. (2006). Scaling and assessment of data quality. Acta Crystallogr D Biol Crystallogr *62*, 72-82.

Hughes, E.N., and August, J.T. (1981). Characterization of plasma membrane proteins identified by monoclonal antibodies. J Biol Chem *256*, 664-671.

Krissinel, E., and Henrick, K. (2007). Inference of macromolecular assemblies from crystalline state. J Mol Biol *372*, 774-797.

Leslie, A.G. (2006). The integration of macromolecular diffraction data. Acta Crystallogr D Biol Crystallogr *62*, 48-57.

McCoy, A.J., Grosse-Kunstleve, R.W., Adams, P.D., Winn, M.D., Storoni, L.C., and Read, R.J. (2007). Phaser crystallographic software. J Appl Crystallogr *40*, 658-674.

Murshudov, G.N., Vagin, A.A., and Dodson, E.J. (1997). Refinement of macromolecular structures by the maximum-likelihood method. Acta Crystallogr D Biol Crystallogr *53*, 240-255.

Peden, A.A., Oorschot, V., Hesser, B.A., Austin, C.D., Scheller, R.H., and Klumperman, J. (2004). Localization of the AP-3 adaptor complex defines a novel endosomal exit site for lysosomal membrane proteins. J Cell Biol *164*, 1065-1076.

Simpson, F., Peden, A.A., Christopoulou, L., and Robinson, M.S. (1997). Characterization of the adaptor-related protein complex, AP-3. J Cell Biol *137*, 835-845.

Steegmaier, M., Klumperman, J., Foletti, D.L., Yoo, J.S., and Scheller, R.H. (1999). Vesicle-associated membrane protein 4 is implicated in trans-Golgi network vesicle trafficking. Mol Biol Cell *10*, 1957-1972.
